# Effects of Zinc-Layered Filler Incorporation Routes on the Antimicrobial, Mechanical, and Physical Properties of Calcium Caseinate Biopolymeric Films

**DOI:** 10.3390/molecules30153307

**Published:** 2025-08-07

**Authors:** Maria E. Becerra, Reynell Pérez-Blanco, Oscar Giraldo, Lucia Medina-Pimentel, Christhy V. Ruiz

**Affiliations:** 1 Laboratorio de Materiales Nanoestructurados y Funcionales, Facultad de Ciencias Exactas y Naturales, Universidad Nacional de Colombia–Sede Manizales, Kilometro 9 vía al Aeropuerto, La Nubia, Manizales 170003, Colombia; maria.becerra@ucaldas.edu.co (M.E.B.); rjperezb@unal.edu.co (R.P.-B.J.); nlmedinap@unal.edu.co (L.M.-P.); cvruizma@unal.edu.co (C.V.R.); 2 Grupo de Investigación en Procesos Químicos, Catalíticos y Biotecnológicos, Universidad Nacional de Colombia-Sede Manizales, Kilometro 9 vía al Aeropuerto, La Nubia, Manizales 170003, Colombia; 3 Departamento de Ingeniería Química, Facultad de Ingeniería y Arquitectura, Universidad Nacional de Colombia-Sede Manizales, Kilometro 9 vía al Aeropuerto, La Nubia, Manizales 170003, Colombia; 4 Departamento de Física y Química, Facultad de Ciencias Exactas y Naturales, Universidad Nacional de Colombia-Sede Manizales, Kilometro 9 vía al Aeropuerto, La Nubia, Manizales 170003, Colombia; 5 Departamento de Química, Universidad de Caldas, Calle 65 No 26-10, Manizales 170001, Colombia

**Keywords:** calcium caseinate films, zinc hydroxide nitrate, nanocomposites, antimicrobial activity, mechanical properties, sustainable food packaging

## Abstract

As the demand for sustainable materials continues to grow, calcium caseinate (Cas) biopolymer films have emerged as promising alternatives to fossil-based plastics. However, their mechanical fragility and high-water sensitivity limit their application in packaging. In this study, we reinforced Cas films with zinc hydroxide nitrate (ZHN) using two incorporation methods: wet (ZHN-w) and dry (ZHN-d). We evaluated how each method affected the dispersion of the filler and, consequently, the functional properties of the films. To our knowledge, this is the first report of ZHN being used in biopolymeric films. Structural and morphological analyses showed better dispersion of ZHN in the wet-incorporated films. These samples exhibited a substantial increase in tensile strength, from 0.75 ± 0.00 MPa to 9.62 ± 2.45 MPa, along with a marked improvement in Young’s modulus. The films also became less soluble in water, more resistant to swelling, and structurally more cohesive. In antimicrobial tests, the ZHN-w films showed stronger inhibition against *E. coli* and *S. aureus*. Overall, this approach offers a simple and effective way to enhance protein-based films using food-safe materials, making them suitable for active and bio-based packaging applications.

## 1. Introduction

Worldwide interest in biodegradable products has initiated considerable development in the plastics sector, especially in the manufacture of polymers based on renewable bio-based feedstocks [1]. This shift aims to minimize the use of fossil feedstocks and create a bio-based economy [2]. Consequently, research efforts have increasingly focused on bio-based polymers and new processes suitable for application in food packaging, thereby creating a sustainable alternative to traditional plastics derived from fossil fuels [3]. Biopolymers, which are biodegradable, renewable, and have a low carbon footprint, are potential alternatives as sustainable food packaging materials [4].

Biopolymers are derived from various sources like polysaccharides, proteins, or lipids, and each category has its own merits. Specifically, proteins are of great importance due to their ready availability, good film-forming characteristics, and high nutritional quality, which makes them suitable for the development of biodegradable films [5]. Protein-based films have better gas barrier characteristics and reduced oxygen permeability compared to polysaccharide- and lipid-based films. Additionally, protein-based films tend to exhibit better mechanical properties compared to those based on polysaccharides and lipids [6].

Among the protein-based films that have been well explored for their suitability in food packaging are those derived from milk, soybeans, fish gelatin, corn zein, and wheat gluten. Proteins derived from animal sources, such as casein, whey protein, collagen, egg whites, and fish myofibrillar proteins, have also found commercial applications [7].

Casein, a highly functional milk protein, possesses an open and flexible structure due to the composition of four main protein types (αS1, αS2, β, and κ) with different characteristics resulting from amino acid sequence differences and post-translational modifications [8]. Sodium caseinate films are generally more flexible [9], while calcium caseinate films are more thermally stable and have improved mechanical properties [10].

Despite their advantages, protein-based materials are inherently brittle, limiting their application in packaging. To address this brittleness, plasticization has been proposed to enhance elasticity, while cross-linking improves tensile strength [11]. However, protein-based biopolymers often require additional reinforcement to meet the mechanical demands of packaging applications. One approach involves incorporating zinc oxide nanoparticles (ZnO-NPs), which not only improve the mechanical properties of biopolymers but also provide antimicrobial benefits due to their ability to generate reactive oxygen species (ROS) [12].

However, the nanometer size and high surface-to-volume ratio of ZnO-NPs present significant challenges [13]. Their propensity to produce ROS can lead to cellular oxidative stress, DNA damage, protein misfolding, and lipid peroxidation, posing a risk of cytotoxicity to human cells and an environmental hazard [14,15]. These potential health risks, even at low levels, limit the use of ZnO-NPs in biopolymer systems [16].

As a safer alternative, clays have been explored as reinforcing materials for biopolymers. Clays do not produce ROS, thereby avoiding the cytotoxicity and environmental risks associated with metal oxide nanoparticles. Research has shown that clays can enhance the physical properties of biopolymeric films, such as vapor permeability, tensile strength, and elongation at break, without the detrimental effects linked to ZnO-NPs. However, while clays significantly improve the mechanical properties of biopolymer films, they lack broad-spectrum antimicrobial activity. Additionally, incompatibility between the biopolymeric matrix and clays can result in clumping, which varies depending on the concentration and dispersion methods [17,18].

Layered materials, such as layered double hydroxides (LDHs) and layered hydroxide salts (LHSs), represent an important class of fillers due to their unique two-dimensional structures, making them suitable for applications in catalysis, separation science, nanocomposite fabrication, and polymer reinforcement [19]. Specifically, zinc hydroxide nitrate (ZHN), a type of layered hydroxide salt with the chemical formula Zn_5_(OH)_8_(NO_3_)_2_.2H_2_O, has emerged as a promising alternative to ZnO nanoparticles due to its structure and functional properties [20]. The ZHN structure consists of a brucite-like layered array of zinc atoms coordinated to OH groups and water molecules, with nitrates in the interlayer spaces serving as charge-balancers [21].

Sulfate, phosphate, and chloride anions are also used to synthesize ZHN type materials [22]. The ability to exchange the interlayer anions in ZHN for other active anions and a diverse range of synthesis pathways provide a wide range of functionalities [23]. For example, ZHN materials intercalated with phosphate anions have been studied for the formulation of controlled-release fertilizers [24]. ZHN has also been investigated as a carrier material for drug delivery with controlled release of bioactive molecules such as 10-hydroxycamptothecin [25], baclofen [26], bispyribac [27], imidacloprid [28], and amoxicillin [29].

Calcination-based oxide routes are widely employed to obtain semiconductor oxides and metallic alloys, because the thermal treatment gives precise control over composition and crystallinity, enabling a versatile range of applications [30]. In this context, ZHN has been used as a calcination precursor that, after thermal conversion, yields ZnO nanostructures capable of photodegrading organic dyes and pharmaceutical pollutants, such as ciprofloxacin, through ROS pathways [31].

Another relevant quality of zinc-containing layered materials is their antimicrobial activity and the associated mechanisms of bacterial inhibition. Several studies have linked the antimicrobial efficacy of zinc-containing layered double hydroxides (Zn-LDHs) against *Escherichia coli* and *Staphylococcus aureus* to (i) electrostatic interactions between their positively charged surfaces and negatively charged bacterial membranes, and (ii) the release of antimicrobial Zn^2+^ ions. The release of Zn^2+^ from Zn-LDHs has been associated with their lower crystallinity, presence of lattice defects, and small aggregate size, which facilitate ion mobility in broth media [32]. In contrast, ROS generation by Zn(OH)_2_, a Zn layered hydroxide material, is lower than that of calcined oxides such as ZnO due to its larger band-gap energy. Wang et al. reported band-gap energies of 3.37 eV for ZnO and 5.65 eV for Zn(OH)_2_, respectively [33]. As a result, the photon energy required to promote the electron–hole pair and initiate ROS generation is significantly higher for Zn(OH)_2_ (219 nm, UV-C region) than for ZnO (368 nm, UV-A region). This suggests that Zn(OH)_2_-based materials, such as ZHN, are unlikely to generate ROS under ambient light, thereby reducing cytotoxic risks while still offering antimicrobial functionality.

In the present study, we propose the inclusion of ZHN as a filler in biopolymeric films made of calcium caseinate, aiming to evaluate its role as a reinforcement and its ability to mitigate the toxicity issues of ZnO-NPs by reducing ROS production. Furthermore, we examine the impact of different incorporation methods on the compatibility and functionality of ZHN within the biopolymeric matrix. This aspect that has not yet been explored in the development of biopolymer films. This approach seeks to provide a safer and more effective alternative for enhancing biopolymeric films used in sustainable packaging.

## 2. Results and Discussion

### 2.1. Biofilm Characterization

Calcium caseinate films were successfully produced using the procedure described in Section 3.2. In parallel, ZHN materials were synthesized according to the methodology reported in Section 3.1, and their details are discussed in the subsequent section. To prepare the calcium caseinate films reinforced with ZHN-w, the moisture content of the ZHN-layered materials was first measured in triplicate following their synthesis. The average moisture content of ZHN was found to be 47.80 ± 9.86%. These values were used to calculate the required amount of dry base material, ensuring consistency with the proposed experimental design.

#### 2.1.1. X-Ray Diffraction Analysis

**Figure 1:**
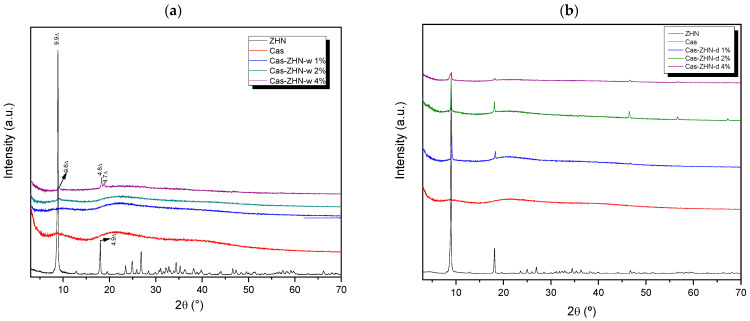
XRD patterns of calcium caseinate films loaded with: (**a**) 1, 2, and 4 wt% of ZHN-w; and (**b**) 1%, 2%, and 4% of ZHN-d.

The XRD patterns of ZHN and calcium caseinate films, as a function of filler concentration and incorporation method, are shown in Figure 1a,b. For the neat calcium caseinate film (Cas), two broad peaks at 2θ = 20° and 2θ = 8.0° indicated the semi-crystalline structure of this biopolymer [34]. According to Colak et al. [35], the reflection at 8.0° and its intensity are associated with the diameter and content of the triple helix in the films. These reflections can be attributed to variations in protein sequences and an asymmetric factor in the primary structure of casein.

The XRD pattern of the filler agent, ZHN, exhibited characteristic reflections typical of layered materials (JCDS 72-0627), with d-values of approximately 9.9 Å and 4.9 Å corresponding to the (200) and (400) diffraction planes, respectively [36]. Minor variations in interlayer spacings were attributed to phases containing higher water content within the layered structure.

In the diffraction patterns of the ZHN-w/Cas nanocomposites (Figure 1a), the (d_200_) diffraction peak disappeared at 1 and 2 wt%, indicating complete dispersion potential exfoliation of the ZHN lattice within the biopolymer matrix [37,38]. However, at a ZHN-w concentration of 4 wt%, reflections appeared at 9.6 Å, 4.8 Å, and 4.7 Å, corresponding to zinc hydroxide layers with lower hydration levels compared to the precursor [36]. Additionally, the shift from 9.9 Å in the pristine ZHN to 9.6 Å in the Cas films containing 4 wt% ZHN-w suggested a slight alteration to the layered structure. This modification may have resulted from interactions between hydrophilic domains in caseinate and ZHN, including hydrogen bonding with the crystalline water in ZHN, which reduced the characteristic crystallization water and narrowed the interlayer spacing.

In contrast, Figure 1b reveals that distinct ZHN reflections persisted across all tested concentrations, suggesting that dry-form ZHN incorporation did not achieve effective dispersion within the Cas matrix.

#### 2.1.2. SEM Studies

**Figure 2:**
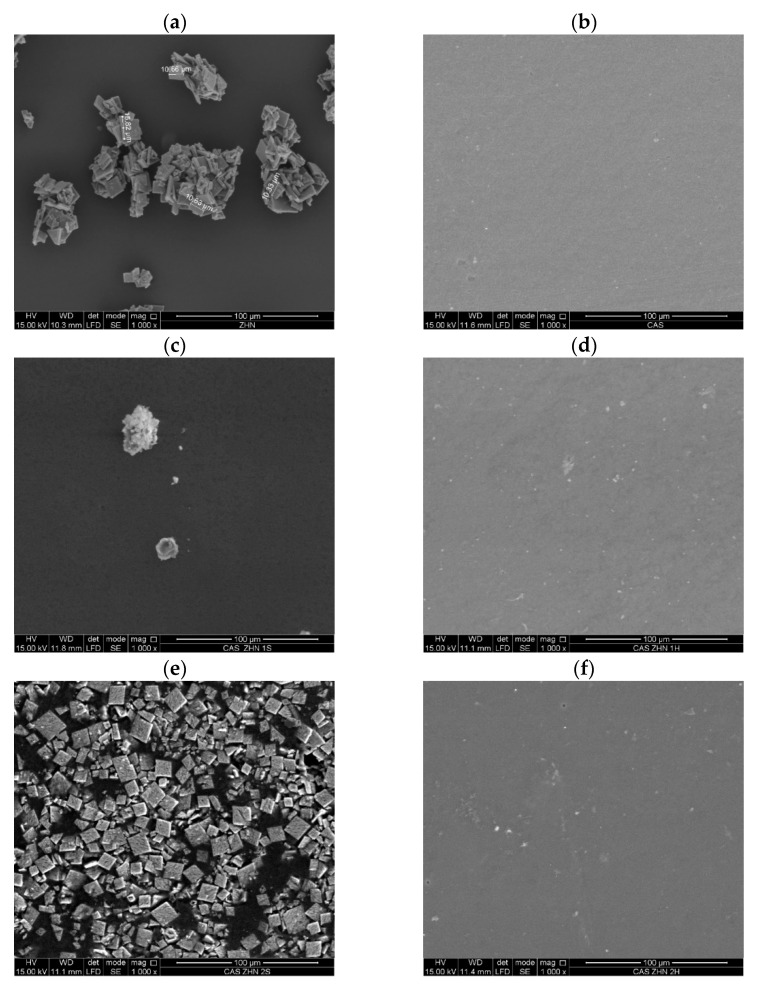

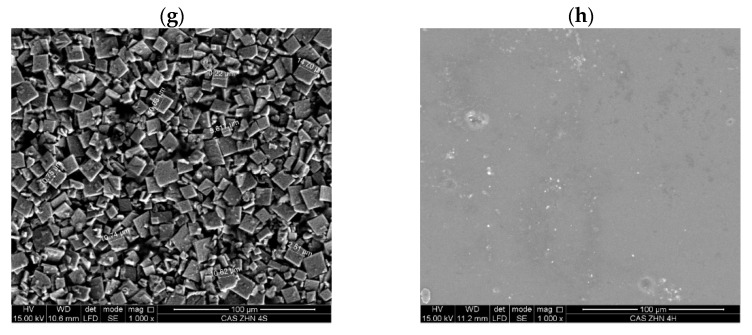
SEM images of surfaces: (**a**) ZHN; (**b**) caseinate films; (**c**) Cas ZHN-d 1%; (**d**) Cas ZHN-w 1%; (**e**) Cas ZHN-d 2%; (**f**) Cas ZHN-w 2%; (**g**) Cas ZHN-d 4%; and (**h**) Cas ZHN-w 4%.

Figure 2 presents SEM images of calcium caseinate films incorporating ZHN via dry and wet routes at different weight percentages. The SEM micrograph of the filler (Figure 2a) reveals the characteristic morphology of rhombohedral, plate-like particles typical of layered materials, with a maximum particle size of 15.62 µm. The control Cas films (Figure 2b) exhibited very small voids, likely caused by trapped air during the drying process [9,39]. However, their surface remained homogeneous and smooth [9].

In contrast, Figure 2c,d illustrate films containing ZHN incorporated through dry and wet methods, respectively. In the dry incorporation route (Figure 2c), ZHN particles were agglomerated on the film surface. Conversely, with the wet incorporation method (Figure 2d), numerous light-colored “dots,” presumably ZHN particles, were dispersed across the surface. The Cas-ZHN-w-1% film exhibited greater surface roughness compared to the neat Cas film.

Figure 2e,f highlight differences in ZHN incorporation routes at 2 wt%. In the dry incorporation route (Figure 2e), the dispersion across the film surface was heterogeneous and irregular, with regions lacking ZHN or localized agglomerations of the filler. In contrast, the wet incorporation method (Figure 2f) displayed no significant changes from the 1 wt% samples, indicating a more uniform dispersion.

Finally, Figure 2g,h depict films with the highest ZHN loadings (4 wt%). In the dry incorporation method (Figure 2g), the film appeared saturated with multi-layered agglomerations of the filler. Conversely, in the wet incorporation method (Figure 2h), minimal differences were observed between the 2 wt% and 4 wt% samples, based on the SEM images.

#### 2.1.3. FT-IR Analysis

**Figure 3:**
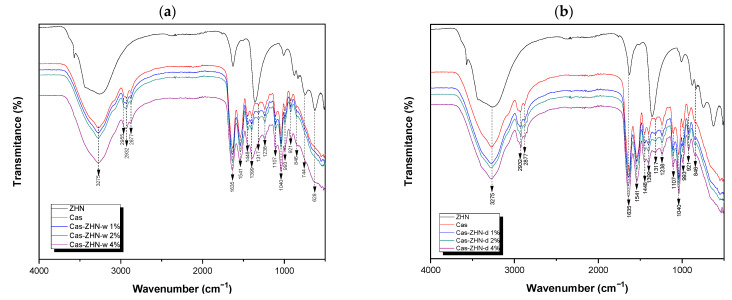
FT-IR spectra of the calcium caseinate films loaded with: (**a**) 1, 2, and 4 wt% of ZHN-w; and (**b**) 1, 2, and 4 wt% of ZHN-d.

Molecular interactions within the films were analyzed using ATR-FTIR (Figure 3). The study included both the raw-material films and those incorporating ZHN as a filler, added in wet and dry forms at various weight percentages relative to the caseinate. Previous research has identified the characteristic bands associated with the layered inorganic structure of ZHN [21].

All composite films exhibited a broad absorption band at approximately 3275 cm^−1^, attributed to hydrogen bonding between the casein matrix and the hydroxyl groups of glycerol, as well as the presence of unbonded NH groups [40]. Additionally, bands at 2955 cm^−1^ and 2932 cm^−1^ were observed, corresponding to the stretching modes of –CH_3_ and –CH_2_ groups, respectively. The band at 2877 cm^−1^ was associated with the symmetric stretching of tertiary C–H bonds [39].

Bands at 1635 cm^−1^ and 1541 cm^−1^ corresponded to the amide I and amide II regions, respectively, reflecting carbonyl group (C=O) stretching and symmetric stretching of N–C=O bonds [9,41,42]. The absorption band at 1448 cm^−1^ was associated with C–H deformation [43], while vibrations around 1399 cm^−1^ indicated the presence of the carboxylate group (O–C–O) [9]. Bands near 1317 cm^−1^ and 1238 cm^−1^ were assigned to the in-plane bending of the hydroxyl group from the plasticizer [9] and the C–OH vibration mode, respectively [44].

Bands at 1107 cm^−1^ and 1040 cm^−1^ corresponded to vibrational frequencies of C–O stretching in C–OH bonds [9,39]. At lower wavenumbers, bands at 993 cm^−1^ and 921 cm^−1^ were ascribed to the dicationic interaction with Ca^2+^ [9].

When comparing the two ZHN incorporation methods (wet and dry), no significant spectral differences were observed at 1 wt% and 2 wt% loadings in the biopolymeric calcium caseinate films. However, at 4 wt% ZHN-w loading, a notable change in the shape of the absorption band and a shift from 1399 cm^−1^ to 1388 cm^−1^ were observed. This shift may be attributed to hydrogen bonding between the carboxylate groups in caseinate proteins and the nitrate anions [40], previously located in the interlaminar region of ZHN, which may have become free at higher concentrations following the intercalation/exfoliation of the filler material.

Additionally, at the highest ZHN-w incorporation (4 wt%), low-wavenumber bands were observed at 744 cm^−1^, corresponding to symmetric stretching vibrations of free nitrate groups within ZHN interlayers [45] and 628 cm^−1^, associated with Zn–OH lattice vibrations within the layers [36]. These findings suggested the coexistence of different forms of the filler material within the biopolymeric film: exfoliated ZHN layers and partially intact layers retaining fewer interlaminar water molecules, as previously suggested by the X-ray diffraction patterns.

### 2.2. Physical Properties of Films

Table 1 summarizes the wettability characteristics of the fabricated films, including moisture content, water solubility, and swelling index.

The moisture content in biopolymeric films is a critical parameter, influencing shelf life, distribution, and transportation [46] due to the permeability of the packaging material. Additionally, moisture exchange between the packaged food product and the film can facilitate microbial growth, depending on the film’s drying parameters and composition [47].

In this study, the film moisture content ranged from 14.05 ± 0.58% to 19.98 ± 2.08% (Table 1), with the highest levels observed in the control (Cas) film compared to the ZHN-reinforced systems. Although moisture content decreased with the addition of ZHN, these differences were not statistically significant (*p* > 0.05) across incorporation routes or concentrations.

The reduction in moisture content could be attributed to changes in the chemical environment of the biopolymeric matrix. These changes resulted from interactions between the functional groups of ZHN and the caseinate protein chains, which decreased the water-binding affinity and reduced the void volume previously occupied by water molecules [48,49].

Since most foods have a water activity above 0.95, the water resistance of a film in high-humidity environments is essential to preventing exudation of fresh or frozen products [50]. Water solubility in biopolymer films plays a critical role in determining their suitability for moist food packaging applications, with their stability being largely dependent on their chemical structure [51,52].

In contrast to the control Cas film, which lost its initial shape during immersion, all ZHN-modified films maintained their integrity after 24 h of incubation in gently stirred water. This observation aligns with the quantitative results in Table 1.

Studies indicate that both protein content and plasticizers in film formulations influence water solubility. For instance, Lam et al. [53] reported reduced solubility with increased sodium caseinate concentration, while Lau et al. [54] found that higher glycerol concentrations in starch and gelatin films enhanced water solubility. Bhatia et al. [50] achieved a water solubility of 32.81 ± 0.62% in calcium caseinate films, whereas Wakai et al. [55] reported 50.90 ± 1.80% for whey protein isolate films.

In comparison, this study’s control film (Cas) exhibited a water solubility of 60.23 ± 5.55%, exceeding previously reported values. This discrepancy likely arose from the distinct proportions of calcium caseinate and glycerol used in the study formulation, which utilized approximately five times more caseinate and four times more glycerol than Bhatia et al. [50]. Such proportions increased the availability of hydroxyl groups [56], which interacted with water molecules to form hydrogen bonds that disrupted the network structure and reduced matrix cohesiveness, thereby increasing water solubility [57].

Regarding ZHN incorporation, adding 1 wt% ZHN-d significantly (*p* < 0.05) reduced the solubility of Cas films, decreasing it from 60.23 ± 5.55% to 44.39 ± 1.55%. Moreover, an inverse relationship was observed between ZHN-d concentration and water solubility.

A distinct trend emerged with ZHN-w: at 1 wt%, water solubility did not change significantly (*p* > 0.05) compared to the control. However, further increases in ZHN-w concentration sharply lowered film solubility compared to the ZHN-d systems. For example, at 2 wt% ZHN, the solubility decreased from 35.35 ± 1.70% (ZHN-d) to 27.05 ± 1.89% (ZHN-w). The lowest water solubility was achieved at 4 wt% ZHN, with a value of 22.27 ± 1.51% in the ZHN-w system. This reduction was attributed to strong hydrogen bonding between the protein chains and hydroxyl groups in the ZHN layers, which enhanced matrix cohesiveness and diminished water solubility.

Additionally, the discrepancy in solubility behavior between dry and wet ZHN methods aligned with XRD and FTIR data. Films containing ZHN-w exhibited lower water solubility compared to their ZHN-d counterparts, suggesting stronger filler–matrix interactions through exfoliation or intercalation. This mechanism increased the contact area between ZHN and protein chains, leading to greater reductions in water solubility compared to non-exfoliated ZHN. SEM images supported this observation: 2 wt% ZHN-d films displayed visible filler saturation on the surface (Figure 2e), whereas ZHN-w films did not display such saturation (Figure 2f).

Numerous authors have reported similar trends in biopolymeric matrices containing clay-type fillers. For example, Wakai et al. [55] attributed the reduced solubility of whey protein isolate films to steric hindrance caused by montmorillonite. Similarly, Hassannia-Kolaee et al. [58] proposed that hydrogen bonds formed between the polymer matrix and clay strengthened the structure, thereby reducing solubility in water. Zolfi et al. [59] also observed lower water solubility in whey protein films containing montmorillonite, attributing this to robust hydrogen bonding among whey protein, kefiran, and the clay, which decreased the water diffusion rates.

The swelling index of biopolymeric films is a critical factor for applications ranging from food packaging to tissue engineering. As shown in Table 1, the control Cas film demonstrated negligible swelling, as it failed to maintain its shape in water. Incorporating ZHN allowed for the modulation of swelling behavior, with swelling indices ranging from 998.32 ± 88.61% to 53.27 ± 8.55%. Generally, higher ZHN loadings significantly decreased swelling capacity (*p* < 0.05), indicating an inverse relationship between filler content and film swellability. This reduction was attributed to reinforced cross-linking via hydrogen bonding between ZHN hydroxyl groups and protein amino [60] or carboxylate [61] moieties, reducing the availability of protein hydroxyl groups for water binding [62].

Significant differences (*p* < 0.05) were also observed between the dry and wet incorporation methods. Films produced with ZHN-d showed lower swelling (53.27 ± 8.55% to 418.36 ± 45.41%) compared to those prepared with ZHN-w (283.68 ± 36.43% to 998.32 ± 88.61%). In the dry incorporation method, the filler material largely retained its crystalline structure, with the host anion fully compensating for the layer’s electrostatic charge. In contrast, the wet route resulted in structural changes, as revealed by XRD and SEM analyses: typical filler morphologies disappeared, and characteristic ZHN diffraction planes were undetected, suggesting an intercalation/exfoliation process. This process separated the host anion and the layers within the biopolymeric matrix, generating electrostatic charges in the caseinate polymer. These charges increased the ionic osmotic pressure, enhancing water uptake as the matrix attempted to counteract the osmotic gradient [63]. Simultaneously, strong hydrogen bonding between the casein network and ZHN particles reduced the film’s water solubility, moisture content, and overall swelling properties.

These results are consistent with previous findings on biopolymeric matrices reinforced with clays and ZnO nanoparticles. Das et al. observed that the addition of montmorillonite to soybean meal protein isolate films reduced their swelling capacity compared to pure biopolymer [64]. Jaberifard et al. reported that halloysite nanotubes (HNTs) significantly decreased the swelling degree of xanthan gum/soy protein-based films as HNT content increased [61]. Similarly, Morariu et al. demonstrated that incorporating Laponite into chitosan films reinforced the biopolymer chains, thereby improving shape integrity during swelling in a concentration-dependent manner [65].

Additionally, Rashidi et al. found that ZnO nanoparticles formed strong hydrogen bonds with the opopanax gum/gelatin network, substantially reducing film swelling [62]. Namazi et al. also observed that adding 1 to 4 wt% ZnO nanoparticles into whey protein films lowered swelling capacity, with the minimum value recorded at the highest ZnO loading [63].

#### Film Transparency

**Figure 4:**
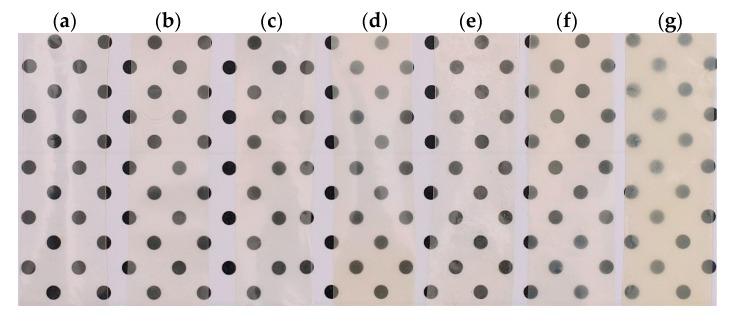
Visual appearance of calcium caseinate films showing the effects of the two routes of ZHN incorporation: (**a**) without filler; Cas-ZHN-w with (**b**) 1%, (**c**) 2%, (**d**) 4%; Cas-ZHN-d with (**e**) 1%, (**f**) 2%, and (**g**) 4%. In both incorporation methods, i.e., wet (w) and dry (**d**), an increase in opacity and coloration of the films was observed as the concentration of ZHN increases.

Transparency is a crucial factor in polymer-based films, as it directly impacts the overall appearance and consumer acceptability [66]. A dot pattern was used as a reference to highlight changes in transparency and coloration of the films resulting from ZHN incorporation. The Cas-ZHN samples displayed a slight whitish hue compared to the control calcium caseinate films. When comparing films with equivalent ZHN-w and ZHN-d percentages (Figure 4), the incorporation of dry ZHN intensified the whitish hue, resulting in greater opacity in films with ZHN-d compared to those with ZHN-w. Additionally, films containing wet ZHN displayed homogeneous and uniform structures without visible irregularities, confirming the good film-forming properties of the biopolymer and its compatibility with the other components.

The results on the transparency of the tested film samples are detailed in Table 2. It was observed that the control sample exhibited maximum transparency compared to the ZHN-Cas film samples. On the other hand, a decrease in the transparency of the ZHN-Cas films was evident as the ZHN concentration increased, both for the dry and wet ZHN addition routes. However, by increasing the concentration of dry ZHN in the biopolymeric matrix, the transparency decreased until reaching a minimum of approximately 20%, unlike what was observed with the incorporation of wet ZHN in the caseinate matrix.

This phenomenon may be related to the transmission of the light beam through the film, where factors such as the addition of a solid agent or the nature of the added agent (such as particle size or dispersion in the biopolymeric matrix) can influence this property [67]. The results obtained by spectrophotometry suggested that the addition of ZHN in the wet state to the caseinate matrix favored a more uniform dispersion or a reduction in particle size compared to the addition of ZHN in the dry state. This hypothesis was reinforced with the SEM images presented above, where the differences in the dispersion and size of the ZHN particles in the caseinate films are evident.

### 2.3. Film Tensile Properties

Polymeric materials, such as films, are subjected to various stresses in their end use, making the determination of their mechanical properties essential from scientific, technological, and practical perspectives [68]. The tensile properties of the Cas and Cas-ZHN films were evaluated by measuring tensile strength and elongation at break.

Initially, a combined statistical analysis of the two experimental blocks (ZHN-d and ZHN-w) was conducted using one-way analysis of variance (ANOVA), followed by post hoc comparisons of means using the Tukey and Dunnett methods. However, residuals did not meet the assumptions of normality or homoscedasticity for all tensile property variables. As a result, each experimental design factor was treated as a randomized block to determine the impact of ZHN concentration on Cas films. Subsequently, a one-way ANOVA was performed, accompanied by post hoc comparisons using the Tukey and Dunnett methods. Additionally, a non-parametric Kruskal-Wallis test was applied to the combined data set to evaluate significant differences stemming from the ZHN incorporation route (wet or dry). Conover-Iman multiple comparisons test was used as post hoc analysis, with the significance threshold set at 5%.

**Figure 5:**
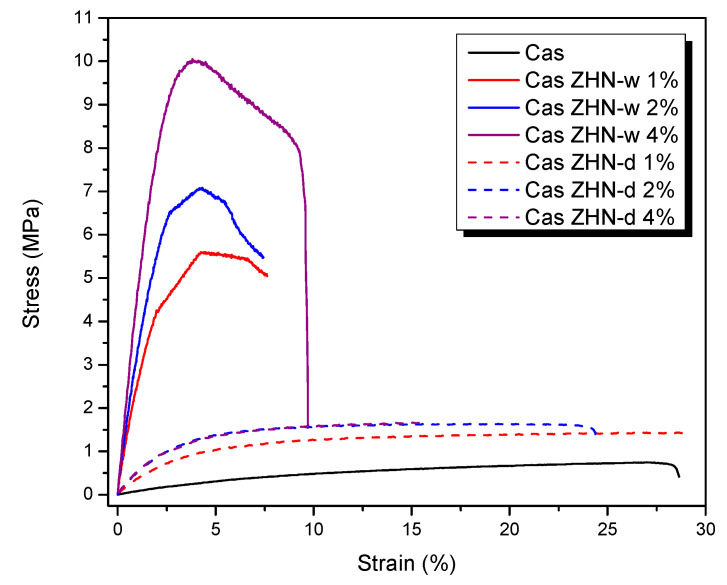
Effect of incorporation route and ZHN concentration on the stress–strain behavior of calcium caseinate-based films. Although seven formulations were tested, only six curves are distinguishable because the Cas-ZHN-d 2% and 4% samples overlap due to their similar mechanical profiles. A slight difference in elongation is observable upon closer inspection.

The tensile properties of the ZHN-reinforced biopolymeric films are summarized in Figure 5. The data revealed that the incorporation of ZHN, irrespective of the incorporation method, significantly enhanced the tensile properties compared to the control film (Cas) (*p* < 0.05). Specifically, adding ZHN to the protein network increased the tensile strength of the biopolymer by at least 42% (*p* < 0.05), while Young’s modulus improved by a factor of at least four (*p* < 0.05) relative to unmodified calcium caseinate films.

When each incorporation route was evaluated separately, it was observed that for the dry incorporation of ZHN in Cas films (Table 3), both tensile strength and Young’s modulus exhibited significant improvements as the filler content increased, while elongation at break showed no statistically significant differences (*p* > 0.05) compared to the control. However, the average elongation at break decreased as the ZHN-d concentration increased.

Table 3 presents the mechanical properties for the dry incorporation route. While the tensile strength and Young’s modulus increased significantly from 1 wt% to 4 wt% ZHN-d, no significant differences were detected between the 2 wt% and 4 wt% additions. This observation aligns with the morphological observations, where micrographs (Figure 2g) show that Cas films reinforced with 2 wt% and 4 wt% ZHN-d reached filler saturation. Consequently, the biopolymer could not effectively accommodate more than 2 wt% ZHN-d without promoting filler agglomeration on the surface. Such an agglomeration can result in localized stress concentrations rather than a uniform stress distribution [69].

The increase in tensile strength and Young’s modulus could be attributed to strong intermolecular interactions between the protein chain functional groups and the hydroxyl groups on the surfaces of the ZHN layers. Furthermore, reductions in water solubility, moisture content, and swelling index in these films suggested robust filler–matrix interactions. These interactions likely induced steric hindrance and reduced the free volume within the biopolymer matrix, thereby leaving fewer active sites available for water molecules. Enhanced cross-linking further strengthened the films, enabling them to endure greater tensile forces [70].

In contrast, Table 4 reveals that wet incorporation (ZHN-w) also resulted in significant increases in tensile strength and Young’s modulus as the ZHN-w concentration increased (*p* < 0.05). However, elongation at break was significantly lower (*p* < 0.05) compared to the control films. Similar trends have been reported for other protein-based films reinforced with clay-like materials or ZnO nanoparticles. For example, Wakai et al. [55] found that adding montmorillonite to whey protein isolate films increased tensile strength from 1.99 MPa to 3.40 MPa while reducing elongation at break from 105.2% to 29.1%. Azevedo et al. [71] demonstrated improvements in tensile strength and Young’s modulus in whey protein isolate films due to cross-linking and exfoliation induced by montmorillonite. Similarly, incorporating ZnO nanoparticles into calcium caseinate biofilms increased tensile strength from 0.49 MPa to 1.65 MPa; this was attributed to strong interfacial interactions between ZnO and the protein matrix [69]. Similar findings have been reported in studies involving nanoclays [72].

In the present study, while the values obtained for ZHN-d were comparable to those reported in the literature, ZHN-w yielded even greater improvements in calcium caseinate films. Specifically, tensile strength increased from 0.75 ± 0.00 MPa to 9.62 ± 2.45 MPa, and Young’s modulus rose from 10.27 ± 0.98 MPa to 489.34 ± 78.29 MPa with the addition of 4 wt% ZHN-w.

This substantial enhancement can be attributed to the dispersion behavior of ZHN-w, which is dependent on its incorporation level. At lower concentrations, dispersion was likely linked to exfoliation, whereas higher filler loadings resulted in a combination of exfoliated and non-exfoliated ZHN. As observed in Cas-ZHN-d, the incorporation of small amounts of ZHN-d enhanced tensile strength by preventing fracture pathways often initiated by intergranular ZHN domains under dry conditions. This phenomenon was supported by SEM micrographs (Figure 2d,f,h) and XRD patterns (Figure 1a).

Furthermore, the differences observed among the 1 wt%, 2 wt%, and 4 wt% ZHN-w group results can be attributed to the mixed state of the filler at higher loadings (Figure 1a). The coexistence of exfoliated and non-exfoliated layers within the biopolymer enhanced molecular interactions among the composite components, further improving tensile performance and reducing film elasticity. Additionally, increased electrostatic interactions within the ZHN layers facilitated cross-linking of the protein chains.

The impact of the incorporation route (wet or dry) on the tensile properties of the reinforced caseinate films was evaluated using the Kruskal-Wallis test (Table 5). Significant differences were observed in tensile strength (K = 19.082, P = 0.004) and Young’s modulus (K = 18.874, P = 0.004). However, elongation at break did not show statistically significant variations among the samples (K = 9.264, P = 0.159).

Post hoc multiple comparisons using the Conover-Iman procedure confirmed that Cas-ZHN-w 4% exhibited the highest tensile strength, with an average value of 9.62 MPa. Additionally, samples containing 2 wt% and 4 wt% ZHN-d, as well as 1 wt%, 2 wt%, and 4 wt% ZHN-w, all showed significant differences (*p* < 0.05) compared to the control. Comparisons between wet and dry incorporation routes at the same filler concentrations revealed that ZHN-w significantly enhanced both tensile strength and Young’s modulus compared to ZHN-d. For example, at a 1 wt% loading, tensile strength rose from 1.31 ± 0.14 MPa (ZHN-d) to 5.47 ± 0.30 MPa (ZHN-w). Similar trends were observed at 2 wt% and 4 wt%.

However, no significant differences (*p* > 0.05) were detected between 2 wt% and 4 wt% ZHN-w or ZHN-d, suggesting that 2 wt% may represent an optimal filler concentration. Overall, the incorporation of ZHN-layered structures substantially improved the mechanical properties of calcium caseinate films, particularly under wet conditions and at higher concentrations.

### 2.4. Antimicrobial Properties

**Figure 6:**
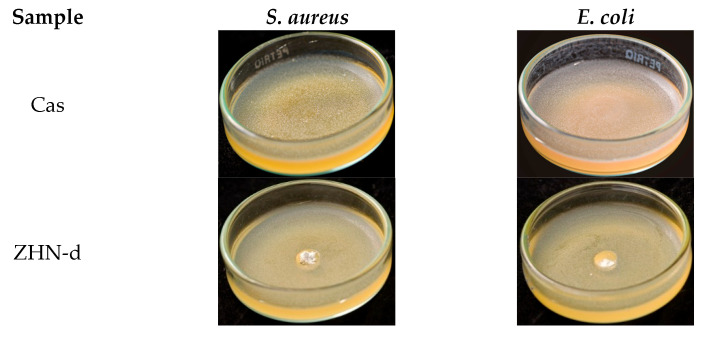

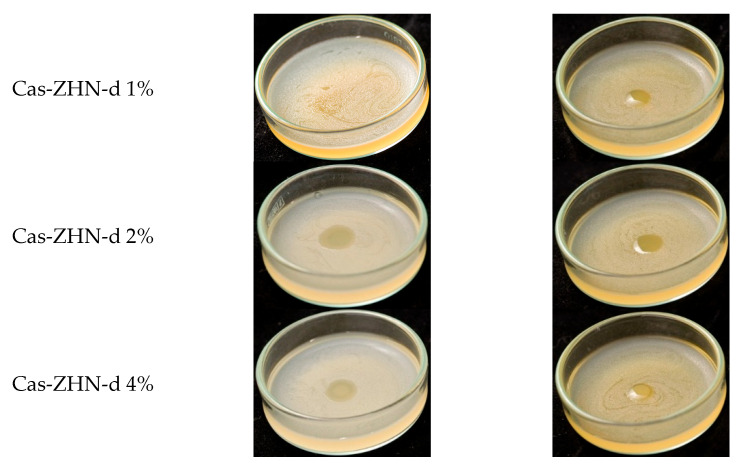
Antimicrobial activity of Cas-ZHN-d system against *S. aureus* and *E. coli*.

**Figure 7:**
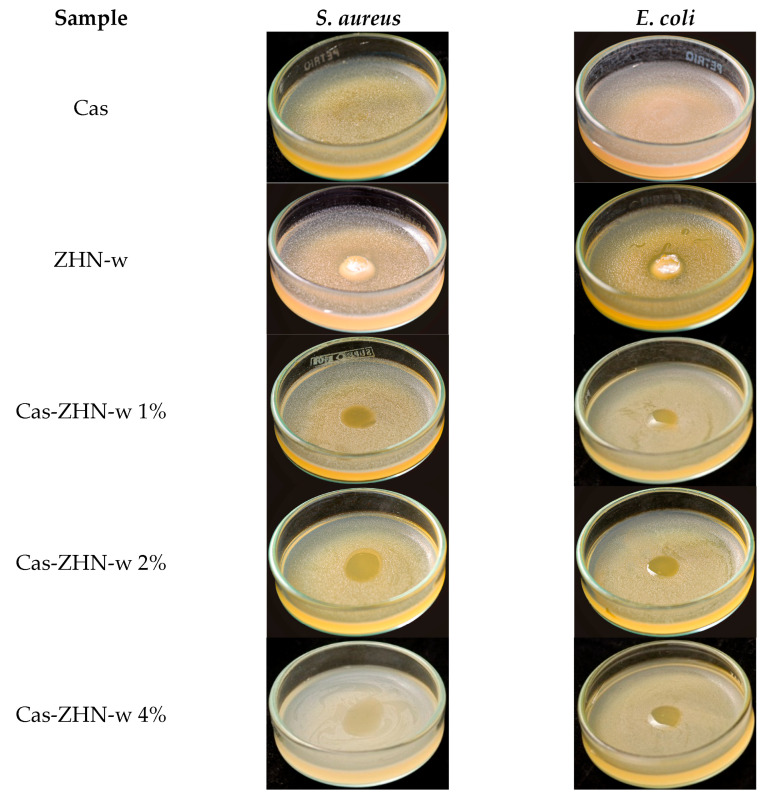
Antimicrobial activity of Cas-ZHN-w system against *S. aureus* and *E. coli*.

The capacity of packaging materials to inhibit bacterial food spoilage or inhibit pathogenic growth is a highly desirable functional attribute [73]. In this study, the antimicrobial activity of Cas and Cas-ZHN nanocomposites against *Escherichia coli* (*E. coli*) and *Staphylococcus aureus* (*S. aureus*) was evaluated using the zone of inhibition method. The findings are detailed in Figure 6 and Figure 7 and Table 6.

As expected, the Cas films exhibited no inhibitory effect. In contrast, the ZHN-layered material demonstrated significant antimicrobial activity, with inhibition zones of 9.3 ± 0.6 mm for *E. coli* and 9.7 ± 0.6 mm for *S. aureus*, highlighting the intrinsic efficacy of ZHN. Despite its promising potential, the available literature on this material remains relatively limited [74]. These results suggest that the antimicrobial activity of Cas-ZHN nanocomposites was closely linked to the presence and mobility of Zn^2+^ ions.

Incorporating ZHN into the casein matrix via the dry route (ZHN-d) showed variable efficiency depending on the concentration (Figure 6). Cas ZHN-d 1% exhibited moderate inhibition (7.0 ± 1.0 mm), i.e., lower than that of ZHN alone but still significantly higher than the control. At 2% ZHN-d, inhibition zones increased significantly (9.7 ± 0.6 mm for *E. coli* and 10.3 ± 0.6 mm for *S. aureus*; *p* < 0.05), indicating that antimicrobial activity correlated with ZHN-d content. However, at 4% ZHN-d, inhibition decreased slightly (7.7 ± 0.6 mm for *E. coli* and 8.3 ± 0.6 mm for *S. aureus*). This decline was likely due to the saturation of ZHN particles within the matrix, as suggested by SEM micrographs (Figure 2e,g) and the reduced water solubility of the films (Table 1). Reduced solubility implies stronger filler–matrix interactions and a more tortuous diffusion path for Zn^2+^ ions, impeding their release and subsequent antimicrobial effect at higher concentrations [75].

Supporting this hypothesis, earlier research on nalidixic acid intercalated into ZHN reported that Zn^2+^ ions exhibit antimicrobial activity against a variety of bacterial and fungal strains [19]. Additionally, Seray et al. [76] reported that Zn^2+^ ions within poly(butylene adipate-co-terephthalate) films containing ZnO nanoparticles significantly disrupt bacterial nutrient transport and enzymatic systems upon contact with the microbial environment.

Table 6 shows that, overall, the nanocomposite films were more effective against gram-positive (*S. aureus*) than gram-negative (*E. coli*), highlighting the importance of cell wall composition [77]. Compared to the dry incorporation route, the wet route (ZHN-w) delivered superior results. Cas ZHN-w 1% showed significantly greater inhibition than Cas ZHN-d 1% (8.7 ± 0.6 mm for *E. coli* and 10.7 ± 0.6 mm for *S. aureus*; *p* < 0.05). At 2% ZHN-w, the inhibition zones reached their maximum (10.3 ± 0.6 mm for *E. coli* and 13.3 ± 0.6 mm for *S. aureus*), indicating optimal filler dispersion and enhanced antimicrobial activity. Increasing the concentration to 4% did not result in further improvement (10.3 ± 0.63 mm for *E. coli* and 14.7 ± 0.6 mm for *S. aureus*), suggesting that 2% ZHN-w may represent the most effective loading. These observations are consistent with the SEM images shown in Figure 2f,h.

Although film solubility decreased with ZHN addition for both incorporation routes, ZHN-w films exhibited greater swelling capacity compared to ZHN-d films. This difference can be attributed to ionic osmotic pressure resulting from electrostatic interactions within the exfoliated and unexfoliated layers, suggesting that Zn^2+^ ions diffused more readily through the wet-incorporated matrix. The increased disc volume observed in Figure 5 and Figure 6 correlates with the films’ swelling capacity and their antimicrobial performance.

Overall, these results demonstrate the efficacy of Cas-ZHN nanocomposites in inhibiting both gram-negative (*E. coli*) and gram-positive (*S. aureus*) bacteria. The improved antimicrobial properties underscore the potential of Cas-ZHN nanocomposites for food packaging applications, particularly in antimicrobial-active packaging systems [78].

## 3. Materials and Methods

### 3.1. Materials

Calcium caseinate powder was generously provided by The Hut Group (THG Company; Manchester, UK). Glycerol (70%) was obtained from Protokimica (Medellín, Colombia). Zinc hydroxide nitrate (ZHN) was prepared using zinc nitrate tetrahydrate [Zn(NO_3_)_2_.4H_2_O; Merck, Macquarie Park, Australia, 98.9%] and zinc oxide (ZnO; Sigma-Aldrich (St. Louis, MI, USA), 99%), following the method described in previous studies [21]. Briefly, 0.09 mol of Zn(NO_3_)_2_.4H_2_O was dissolved in 60.0 mL of deionized water (DW), and 0.09 mol of ZnO was dispersed in a separate 60.0 mL aliquot of DW. The two suspensions were combined and stirred at 400 rpm for 24 h at room temperature (20 ± 1 °C). The resulting solid was collected by filtration and washed three times with DW.

### 3.2. Preparation of Calcium Caseinate Biofilms

The casting solutions were prepared by following the synthesis method reported by Arrieta [9], with slight modifications. Initially, 5000 g of calcium caseinate (Cas) was dissolved in 100.0 mL of DW, resulting in a 5% (*w*/*v*) solution. The solution was mixed at 400 rpm for 10 min. Then, 2.0 mL of glycerol was added as a plasticizer with constant mechanical stirring. Two routes were employed for incorporating zinc-layered materials into the films: the first involved replacing 1%, 2%, and 4% by weight of the wet ZHN, while the second used dry ZHN at the same percentages.

The resulting dispersions were divided, and each part was poured into a square mold (12 × 12 cm). The films were dried at 20 ± 1 °C and 70 ± 1% relative humidity until complete solvent evaporation, which typically occurred within three days. After drying, the films were stored at room temperature until characterization.

### 3.3. Experimental Design

A 2^3^ factorial design was employed to investigate the effects of ZHN incorporation into calcium caseinate films. Two incorporation methods, using wet ZHN (ZHN-w) and dry ZHN (ZHN-d), were considered as independent factors. Each factor included three concentration levels of ZHN (1%, 2%, and 4% by weight relative to calcium caseinate). The response variables included transparency, elongation, strength, Young’s modulus, solubility, and antimicrobial properties.

### 3.4. Biofilms Characterization

#### 3.4.1. X-Ray Diffraction

X-ray diffraction (XRD) analysis was conducted using a MiniFlex II diffractometer (Rigaku Holdings Corporation, Tokyo, Japan). The instrument operated at a voltage of 30 kV and a current of 15 mA. Diffraction data were collected across 2θ angles ranging from 3° to 70°, with a scan rate of 1.5° per minute and a step size of 0.02°.

#### 3.4.2. ATR-FTIR Spectroscopy

Chemical groups and bonding patterns of the synthesized sample components were analyzed using attenuated total reflectance-Fourier transform infrared spectroscopy (ATR-FTIR) with an infrared spectrometer (Alpha Platinum-ATR, Bruker; Billerica, MA, USA). Spectra were collected at a resolution of 4 cm^−1^, ranging from 4000 cm^−1^ to 500 cm^−1^.

#### 3.4.3. Scanning Electron Microscopy (SEM)

Scanning electron micrographs were obtained using a JSM5910LV microscope (JEOL, Tokyo, Japan), equipped with secondary electron (SEI) detectors, operated at 15 kV in high vacuum mode.

### 3.5. Physical Properties

#### 3.5.1. Moisture Content

Moisture content (MC) was measured using 20 mm diameter discs, which were dried at 105 °C until a constant weight was achieved [79]. MC was expressed as the percentage ratio of loss of weight to the initial sample weight.

#### 3.5.2. Swelling Index (%)

For swelling studies [79], dry discs (10 mm in diameter) were immersed in beakers containing 50 mL of water and 0.01% sodium azide to prevent casein spoilage, at 25 °C. The discs were removed at different intervals, superficially dried with tissue paper, weighed, and then returned to the water. The process was repeated until constant weight was achieved. The degree of swelling (DS) was calculated using Equation (1), where W_s_ and W_d_ represent the weights of the swollen and dry samples, respectively.

(1) $$DS=\frac{W_{s}-W_{d}}{W_{d}}\times 100$$

#### 3.5.3. Solubility Index (%)

The solubility test followed the methodology proposed by Gontard et al. [80], with slight modifications. Solubility was expressed as the percentage of dry matter from the film solubilized after 24 h of immersion in water. The initial dry matter content of each film was determined at 70 °C for 72 h. First, 0.50 ± 0.05 g of film was weighed, submerged in 50.0 mL of DW containing traces of sodium azide (0.02% *w*/*v*) to prevent microorganism growth, and stirred in an electronic shaker (Mazzine TA-09E, INDULAB, Area de Promoción El Triángulo, Argentina) at 130 rpm for 24 h at 20 ± 5 °C. The film pieces were subsequently removed and dried at 70 °C for 24 h to determine the weight of dry matter not solubilized in water. The percentage of solubilized dry matter (%Sol) was calculated following Equation (2) by subtracting the weight of non-solubilized dry matter (W_sol_) from the initial dry matter weight (W_Idry_), and reported based on the initial dry weight:

(2) $$\%\text{Sol}=\frac{W_{\text{Idry}}-W_{\text{Sol}}}{W_{\text{Idry}}}*100$$

#### 3.5.4. Transparency Film

For the transparency test, Cas biofilms were cut into rectangular shapes (20 × 50 mm) and fixed on the outer side of a cuvette. The cuvette was placed in a vis-spectrophotometer (GENESYS 30, Thermo Scientific; Waltham, MA, USA) with the film closest to the light beam. Absorbance (A) at 600 nm was used to calculate transparency (%T) according to the ASTM standard in Equation (3) [81]. These experimental measurements were based on the results obtained by Zhao et al. [82].

(3) $$\%T=10^{2-A}$$

### 3.6. Film Tensile Properties

Tensile testing was conducted at room temperature (20 ± 1 °C) using an ESM303 universal testing machine (Mark-10, Copiague, NY, USA), which featured a displacement resolution of 0.02 mm and a load cell resolution of 0.01 mm. Rectangular specimens, measuring 2.5 cm in width and 10 cm in length, were cut from the films. These specimens were securely clamped in film tensile grips and stretched at a constant speed of 3 mm/min until failure, following the standard test method outlined in ASTM D882-18 [83]. The average film thickness was incorporated into the calculation of tensile properties.

### 3.7. Antimicrobial Properties of Synthesized Materials

The target microorganisms for evaluating antibacterial activity were *E. coli* (ATCC 25922) and *S. aureus* (ATCC 25923). Bacterial cultures were preactivated by overnight incubation at 37 °C on Mueller-Hinton (M-H) broth. A bacterial suspension was prepared in sterile saline solution and adjusted to a turbidity equivalent of 0.5 on the McFarland scale (≈1.5 × 10^8^ CFU/mL), using a spectrophotometer at 546 nm.

For each assay, 10 mL of M-H agar was melted, cooled to 55 °C, and inoculated with 0.5 mL of the bacterial suspension before being poured into 55 mm × 15 mm Petri dishes on a leveled surface. The antibacterial activity of the synthesized powder materials was assessed using the agar well diffusion method [84], while the antibacterial activity of films was evaluated using the agar disk diffusion assay [85].

For the well diffusion assay, once the medium solidified, a 5 mm diameter well was cut from the agar. Then, 25 mg of the material was placed into the well, followed by 50 µL of sterile saline solution to facilitate diffusion.

For the disk diffusion assay, films were cut into 6.0 ± 0.1 mm diameter disks and exposed to ultraviolet light for 1 h. The disinfected disks were placed on the M-H agar surface.

After 24 h of incubation at 37 °C, the plates were examined for clear zones around the wells or films, indicating antimicrobial activity. An aqueous gentamicin solution (100 mg/mL, Sigma-Aldrich) was used as the positive control (50 µL per well). The results were reported as the diameter (mm) of the inhibition zones.

### 3.8. Statistical Analysis

Statistical analysis was performed using XLSTAT software (https://www.xlstat.com (accessed on 23 June 2024)) [86]. Assumptions of normality, homoscedasticity, and independence were verified using the Shapiro-Wilk test, Levene’s test, and graphical plots, respectively.

Parametric analysis of variance (ANOVA) was conducted to identify significant differences in film properties attributed to the addition of ZHN. Post hoc tests, including Tukey’s and Dunnett’s tests, were applied to detect significant differences between test groups and the control group, with a confidence level of 95%.

When the assumptions of normal distribution and equal variances were not met, a non-parametric Kruskal-Wallis test was employed to assess significant differences in properties due to ZHN inclusion. Additionally, pooled data from both conditions were analyzed to determine the effects of adding ZHN in wet or dry form. For post hoc analysis, Conover-Iman’s multiple comparisons test was utilized, with a significance level of 5%.

All assays were performed in triplicate for each characterization method. The experimental design followed a 2 × 3 factorial scheme, which included three ZHN concentrations (1%, 2%, and 4%) for each incorporation route (wet and dry), along with the control (Cas) sample. All results are reported as mean ± standard deviation.

## 4. Conclusions

In this study, we explored the use of zinc hydroxide nitrate (ZHN) to improve the properties of calcium caseinate-based films. What stood out most was the influence of the incorporation method. When ZHN was added in wet form, the films became not only stronger and stiffer but also less soluble and more resistant to swelling. These improvements are consistent with better dispersion and closer interaction between the filler and the protein matrix, as observed in the morphological and structural analyses.

We also observed that ZHN-w films offered greater antimicrobial activity compared to their dry counterparts, which we attribute to enhanced Zn^2+^ ion release. Considering the simplicity of the synthesis and the food-grade nature of the components used, this approach presents a practical way to develop active, bio-based films. While our study did not assess biodegradability directly, the materials used suggest compatibility with environmentally conscious applications.

This is, to our knowledge, the first report using ZHN in biopolymeric films. Future work could investigate the behavior of these films under real packaging conditions or in combination with other functional additives.

## Figures and Tables

**Table 1:** Mean values for moisture content, swelling index, and water solubility of calcium caseinate films with and without ZHN as a filler material, via dry (d) and wet (w) incorporation routes.

Sample	Moisture Content (%)	Swelling Index (%)	Water Solubility (%)
Cas	19.98 ± 2.08 ^b^	*	60.23 ± 5.55 ^d^
Cas ZHN-d 1%	15.63 ± 0.57 ^a^	418.37 ± 45.41 ^c^	44.39 ± 1.55 ^c^
Cas ZHN-d 2%	15.64 ± 1.17 ^a^	163.06 ± 19.52 ^ab^	35.35 ± 1.70 ^b^
Cas ZHN-d 4%	14.21 ± 0.84 ^a^	53.27 ± 8.55 ^a^	34.80 ± 1.24 ^b^
Cas ZHN-w 1%	14.83 ± 1.47 ^a^	998.32 ± 88.61 ^e^	65.22 ± 4.56 ^d^
Cas ZHN-w 2%	14.05 ± 0.58 ^a^	589.65 ± 58.85 ^d^	27.05 ± 1.89 ^ab^
Cas ZHN-w 4%	14.82 ± 0.90 ^a^	283.68 ± 36.43 ^bc^	22.27 ± 1.51 ^a^

* The result could not be quantified due to its high solubility. The ± sign means standard deviation. The values with different letters (a, b, c, d, and e) within a column indicate significant differences (*p* < 0.05).

**Table 2:** Mean values of film transparency of calcium caseinate films with and without ZHN as a filler material, according to the dry and wet incorporation routes (d and w).

Sample	Transparency (%)
Cas	90.303 ± 1.462 ^e^
Cas ZHN-d 1%	83.898 ± 2.000 ^de^
Cas ZHN-d 2%	62.620 ± 3.252 ^b^
Cas ZHN-d 4%	20.559 ± 4.417 ^a^
Cas ZHN-w 1%	87.015 ± 3.667 ^de^
Cas ZHN-w 2%	79.515 ± 2.229 ^cd^
Cas ZHN-w 4%	70.427 ± 4.981 ^bc^

The ± sign means standard deviation. The values with different letters (a, b, c, d, and e) within a column indicate significant differences (*p* < 0.05).

**Table 3:** Averages for tensile strength, maximum strain, and Young’s modulus, with standard deviations and Tukey categories for Cas films incorporating ZHN-d.

Sample	Tensile Strength (MPa)	Strain (%)	Young’s Modulus (MPa)
Cas	0.75 ± 0.00 ^a^	29.70 ± 1.02 ^a^	10.27 ± 0.98 ^a^
Cas ZHN-d 1%	1.31 ± 0.14 ^b^	15.23 ± 12.05 ^a^	42.09 ± 12.10 ^b^
Cas ZHN-d 2%	1.61 ± 0.16^c^	14.50 ± 8.71 ^a^	61.52 ± 7.73 ^c^
Cas ZHN-d 4%	1.74 ± 0.07 ^c^	10.70 ± 4.16 ^a^	68.01 ± 6.38 ^c^

The ± sign means standard deviations. The values with different letters (a, b and c) within each column differ significantly (*p* < 0.05).

**Table 4:** Averages for tensile strength, maximum strain, and Young’s modulus, with standard deviations and Tukey categories for Cas films incorporating ZHN-w.

Sample	Tensile Strength (MPa)	Strain (%)	Young’s Modulus (MPa)
Cas	0.75 ± 0.00 ^a^	29.70 ± 1.02 ^b^	10.27 ± 0.98 ^a^
Cas ZHN-w 1%	5.47 ± 0.30 ^b^	11.00 ± 2.96 ^a^	325.75 ± 34.37 ^b^
Cas ZHN-w 2%	6.45 ± 0.89 ^b^	8.17 ± 1.78 ^a^	341.10 ± 38.52 ^b^
Cas ZHN-w 4%	9.62 ± 2.45 ^c^	10.50 ± 0.80 ^a^	489.34 ± 78.29 ^c^

The ± sign means standard deviations. The values with different letters (a, b, and c) within each column differ significantly (*p* < 0.05).

**Table 5:** Average tensile strength, elongation at break, and Young’s modulus, with standard deviations and Conover-Iman categories, for Cas films with ZHN according to incorporation route.

Sample	Tensile Strength (MPa)	Elongation at Break (%)	Young’s Modulus (MPa)
Cas	0.75 ± 0.00 ^f^	29.70 ± 1.02	10.27 ± 0.98 ^d^
Cas ZHN-d 1%	1.31 ± 0.14 ^e^	15.23 ± 12.05	42.09 ± 12.10 ^cd^
Cas ZHN-d 2%	1.61 ± 0.16 ^de^	14.50 ± 8.71	61.52 ± 7.73 ^c^
Cas ZHN-d 4%	1.74 ± 0.07 ^cd^	10.70 ± 4.16	68.01 ± 6.38 ^bc^
Cas ZHN-w 1%	5.47± 0.30 ^bc^	11.00 ± 2.96	325.75 ± 34.37 ^ab^
Cas ZHN-w 2%	6.45 ± 0.89 ^ab^	8.17 ± 1.78	341.10 ± 38.52 ^a^
Cas ZHN-w 4%	9.62 ± 2.45 ^a^	10.50 ± 0.80	489.34 ± 78.29 ^a^

The ± sign means standard deviations. The values with different letters (a, b, c, d, e, and f) within each column differ significantly (*p* < 0.05).

**Table 6:** Average of inhibition halo against *E. coli* and *S. aureus*, with standard deviations and Tukey’s categories of the Cas films with ZHN according to the wet incorporation route.

Sample	*E. coli* (mm)	*S. aureus* (mm)
Cas	0.0 ± 0.0 ^a^	0.0 ± 0.0 ^a^
ZHN	9.3 ± 0.6 ^cd^	9.7 ± 0.6 ^cd^
Cas ZHN-d 1%	7.0 ± 1.0 ^b^	7.0 ± 1.0 ^b^
Cas ZHN-d 2%	9.7 ± 0.6 ^d^	10.3 ± 0.6 ^d^
Cas ZHN-d 4%	7.7 ± 0.6 ^bc^	8.3 ± 0.6 ^bc^
Cas ZHN-w 1%	8.7 ± 0.6 ^bcd^	10.7 ± 0.6 ^d^
Cas ZHN-w 2%	10.3 ± 0.6 ^d^	13.3 ± 0.6 ^e^
Cas ZHN-w 4%	10.3 ± 0.63 ^d^	14.7 ± 0.6 ^e^

The ± sign means standard deviations. The values with different letters (a, b, c, d, and e) within a column indicate significant differences (*p* < 0.05).

## Data Availability

The original contributions presented in this study are included in the article. Further inquiries can be directed to the corresponding author.

## Abbreviations

The following abbreviations are used in this manuscript:

%Sol	percentage of solubilized dry matter
ANOVA	analysis of variance
ASTM	American Society for Testing and Materials
ATCC	American Type Culture Collection
ATR-FTIR	attenuated total reflectance-Fourier transform infrared
Cas	Calcium caseinate
CFU	Colony Forming Units
cm	centimeter
DNA	deoxyribonucleic acid
DS	degree of swelling
DW	Deionized water
*E. coli*	*Escherichia coli*
HNTs	halloysite nanotubes
JCDS	Joint Committee on Powder Diffraction Standards
kV	kilo volts
LDHs	layered double hydroxides
LHSs	layered hydroxide salts
MC	Moisture content
mg	milligrams
min	minute
mA	milliamps
mm	millimeters
mL	milliliters
MPa	megapascals
MH	Mueller-Hinton
nm	nanometers
ROS	reactive oxygen species
*S. aureus*	*Staphylococcus aureus*
SEI	secondary electron imaging
SEM	scanning electron microscopy
THG	The Hut Group Company
Wd	weight of the dry samples
WIdry	weight of initial dry matter
Ws	weight of the swollen samples
Wsol	weight of non-solubilized dry matter
wt%	weight percent
XRD	X-ray diffraction
ZHN	zinc hydroxide nitrate
ZHN-d	dry zinc hydroxide nitrate
ZHN-w	wet zinc hydroxide nitrate
ZnO-NPs	zinc oxide nanoparticles
µL	microliters
Å	angstrom units

## References

[1] Babaei-Ghazvini A., Acharya B., Korber D.R. (2021). Antimicrobial Biodegradable Food Packaging Based on Chitosan and Metal/Metal-Oxide Bio-Nanocomposites: A Review. Polymers.

[2] Asgher M., Qamar S.A., Bilal M., Iqbal H.M. (2020). Bio-based active food packaging materials: Sustainable alternative to conventional petrochemical-based packaging materials. Food Res. Int..

[3] Wang S., Chen H., Tong Y., Li Y., Zhang J., Chen C., Ren F., Hou C., Wang P. (2023). Composite films with properties improved by increasing the compatibility of sodium caseinate and zein in a heated 60% ethanol solvent. Food Hydrocoll..

[4] Zubair M., Ullah A. (2020). Recent advances in protein derived bionanocomposites for food packaging applications. Crit. Rev. Food Sci. Nutr..

[5] Zioga M., Papantonopoulou G., Evageliou V. (2023). High internal phase emulsions and edible films with high methoxyl pectin and pea protein isolate or sodium caseinate. Food Hydrocoll..

[6] Chen H., Wang J., Cheng Y., Wang C., Liu H., Bian H., Pan Y., Sun J., Han W. (2019). Application of Protein-Based Films and Coatings for Food Packaging: A Review. Polymers.

[7] Arora A., Padua G. (2010). Review: Nanocomposites in Food Packaging. J. Food Sci..

[8] Casanova F., Nascimento L.G.L., Silva N.F., de Carvalho A.F., Gaucheron F. (2021). Interactions between caseins and food-derived bioactive molecules: A review. Food Chem..

[9] Arrieta M.P., Peltzer M.A., Garrigós M.d.C., Jiménez A. (2013). Structure and mechanical properties of sodium and calcium caseinate edible active films with carvacrol. J. Food Eng..

[10] Belyamani I., Prochazka F., Assezat G., Debeaufort F. (2014). Mechanical and barrier properties of extruded film made from sodium and calcium caseinates. Food Packag. Shelf Life.

[11] Lamp A., Kaltschmitt M., Dethloff J. (2022). Options to Improve the Mechanical Properties of Protein-Based Materials. Molecules.

[12] Reda A.T., Park J.Y., Park Y.T. (2024). Zinc Oxide-Based Nanomaterials for Microbiostatic Activities: A Review. J. Funct. Biomater..

[13] Vimbela G.V., Ngo S.M., Fraze C., Yang L., Stout D.A. (2017). Antibacterial properties and toxicity from metallic nanomaterials. Int. J. Nanomed..

[14] Kang M., Liu Y., Weng Y., Wang H., Bai X. (2024). A critical review on the toxicity regulation and ecological risks of zinc oxide nanoparticles to plants. Environ. Sci. Nano.

[15] Nel A., Xia T., Maädler L., Li N. (2006). Toxic Potential of Materials at the Nanolevel. Science.

[16] Kumah E.A., Fopa R.D., Harati S., Boadu P., Zohoori F.V., Pak T. (2023). Human and environmental impacts of nanoparticles: A scoping review of the current literature. BMC Public Health.

[17] Perera K.Y., Hopkins M., Jaiswal A.K., Jaiswal S. (2024). Nanoclays-containing bio-based packaging materials: Properties, applications, safety, and regulatory issues. J. Nanostructure Chem..

[18] Zeggai F.Z., Touahra F., Labied R., Lerari D., Chebout R., Bachari K. (2024). Biopolymers-Clay Nanocomposites: Synthesis Pathways, Properties, and Applications.

[19] Nabipour H., Sadr M.H., Thomas N. (2016). Synthesis, controlled release and antibacterial studies of nalidixic acid–zinc hydroxide nitrate nanocomposites. New J. Chem..

[20] Hahsim N., Muda Z., Isa I.M., Abu Bakar N., Mahamod W.R.W., Ali N.M., Sharif S.N.M., Jajuli M.N., Zobir S.A.M., Suyanta S. (2023). Synthesis and Application of Zinc Layered Hydroxide: A Short Review. Indones. J. Chem..

[21] Ruiz C.V., Rodríguez-Castellón E., Giraldo O. (2018). Structural Analysis and Conduction Mechanisms in Polycrystalline Zinc Hydroxide Nitrate. Inorg. Chem..

[22] Shinagawa T., Watanabe M., Mori T., Tani J.-I., Chigane M., Izaki M. (2018). Oriented Transformation from Layered Zinc Hydroxides to Nanoporous ZnO: A Comparative Study of Different Anion Types. Inorg. Chem..

[23] da Gama B.M.V., Selvasembian R., Giannakoudakis D.A., Triantafyllidis K.S., McKay G., Meili L. (2022). Layered Double Hydroxides as Rising-Star Adsorbents for Water Purification: A Brief Discussion. Molecules.

[24] Khadiran N.F., Hussein M.Z., Ahmad R., Khadiran T., Zainal Z., Kadir W.R.W.A., Hashim S.S. (2021). Preparation and properties of zinc layered hydroxide with nitrate and phosphate as the counter anion, a novel control release fertilizer formulation. J. Porous Mater..

[25] Zhang Y., Mi Y., Liu M., Zeng S., Hou W. (2024). Synthesis of (10-hydroxycamptothecin intercalated layered zinc hydroxide nitrate)@liposome nanocomposites for improving drug-release performance. J. Mol. Liq..

[26] Nabipour H., Batool S., Hu Y. (2023). Pectin-Coated Baclofen-Layered Zinc Hydroxide Nanohybrid as a Bio-Based Nanocomposite Carrier for Oral Delivery. IEEE Trans. Nanobioscience.

[27] Sharif S.N.M., Hashim N., Isa I.M., Bakar S.A., Saidin M.I., Ahmad M.S., Mamat M., Hussein M.Z., Zainul R. (2021). Chitosan as a coating material in enhancing the controlled release behaviour of zinc hydroxide nitrate–sodium dodecylsulphate–bispyribac nanocomposite. Chem. Pap..

[28] Sharif S.N.M., Hashim N., Isa I.M., Abu Bakar S., Saidin M.I., Ahmad M.S., Mamat M., Hussein M.Z., Zainul R. (2020). The impact of a hygroscopic chitosan coating on the controlled release behaviour of zinc hydroxide nitrate–sodium dodecylsulphate–imidacloprid nanocomposites. New J. Chem..

[29] Nabipour H., Sadr M.H., Thomas N. (2015). Synthesis, characterisation and sustained release properties of layered zinc hydroxide intercalated with amoxicillin trihydrate. J. Exp. Nanosci..

[30] Trukhanov A.V., Darwish K.A., Salem M.M., Hemeda O.M., Abdel Ati M.I., Darwish M.A., Kaniukov E.Y., Podgornaya S.V., Turchenko V.A., Tishkevich D.I. (2021). Impact of the heat treatment conditions on crystal structure, morphology and magnetic properties evolution in BaM nanohexaferrites. J. Alloys Compd..

[31] Ibn Mahrsi M., Chouchene B., Gries T., Carré V., Girot E., Medjahdi G., Ayari F., Balan L., Schneider R. (2023). Novel ZnO/Ag nanohybrids prepared from Ag^+^-doped layered zinc hydroxides as highly active photocatalysts for the degradation of dyes and Ciprofloxacin. Colloids Surf. A Physicochem. Eng. Asp..

[32] Awassa J., Cornu D., Soulé S., Carteret C., Ruby C., El-Kirat-Chatel S. (2022). Divalent metal release and antimicrobial effects of layered double hydroxides. Appl. Clay Sci..

[33] Wang M., Jiang L., Kim E.J., Hahn S.H. (2015). Electronic structure and optical properties of Zn(OH)_2_: LDA^+^U calculations and intense yellow luminescence. RSC Adv..

[34] Ranjbaryan S., Pourfathi B., Almasi H. (2019). Reinforcing and release controlling effect of cellulose nanofiber in sodium caseinate films activated by nanoemulsified cinnamon essential oil. Food Packag. Shelf Life.

[35] Colak B.Y., Gouanve F., Degraeve P., Espuche E., Prochazka F. (2015). Study of the influences of film processing conditions and glycerol amount on the water sorption and gas barrier properties of novel sodium caseinate films. J. Memb. Sci..

[36] Ruiz C.V., Giraldo O. (2019). Evaluation of the electrical and dielectric behavior of hybrid materials based on layered zinc hydroxide and benzoate. Ionics.

[37] Ludueña L.N., Vázquez A., Alvarez V.A. (2013). Effect of the type of clay organo-modifier on the morphology, thermal/mechanical/impact/barrier properties and biodegradation in soil of polycaprolactone/clay nanocomposites. J. Appl. Polym. Sci..

[38] Nejad H.M., Ganster J., Volert B. (2010). Starch esters with improved mechanical properties through melt compounding with nanoclays. J. Appl. Polym. Sci..

[39] Pereda M., Aranguren M.I., Marcovich N.E. (2008). Characterization of chitosan/caseinate films. J. Appl. Polym. Sci..

[40] Mohamed A., Ramaswamy H.S. (2022). Characterization of Caseinate–Carboxymethyl Chitosan-Based Edible Films Formulated with and without Transglutaminase Enzyme. J. Compos. Sci..

[41] Eckard A.D., Muthukumarappan K., Gibbons W. (2012). Analysis of Casein Biopolymers Adsorption to Lignocellulosic Biomass as a Potential Cellulase Stabilizer. J. Biomed. Biotechnol..

[42] Pereda M., Amica G., Rácz I., Marcovich N.E. (2011). Structure and properties of nanocomposite films based on sodium caseinate and nanocellulose fibers. J. Food Eng..

[43] Barreto P., Pires A., Soldi V. (2003). Thermal degradation of edible films based on milk proteins and gelatin in inert atmosphere. Polym. Degrad. Stab..

[44] Cardoso J.C., Albuquerque R.L.C., Padilha F.F., Bittencourt F.O., de Freitas O., Nunes P.S., Pereira N.L., Fonseca M.J.V., Araújo A.A.S. (2011). Effect of the Maillard reaction on properties of casein and casein films. J. Therm. Anal. Calorim..

[45] Ruiz C.V., Rodríguez-Castellón E., Giraldo O. (2019). Hybrid materials based on a layered zinc hydroxide solid and gallic acid: Structural characterization and evaluation of the controlled release behavior as a function of the gallic acid content. Appl. Clay Sci..

[46] Vahedikia N., Garavand F., Tajeddin B., Cacciotti I., Jafari S.M., Omidi T., Zahedi Z. (2019). Biodegradable zein film composites reinforced with chitosan nanoparticles and cinnamon essential oil: Physical, mechanical, structural and antimicrobial attributes. Colloids Surf. B Biointerfaces.

[47] Bonnaillie L.M., Zhang H., Akkurt S., Yam K.L., Tomasula P.M. (2014). Casein Films: The Effects of Formulation, Environmental Conditions and the Addition of Citric Pectin on the Structure and Mechanical Properties. Polymers.

[48] Hasheminya S.-M., Dehghannya J. (2021). Development and characterization of novel edible films based on Cordia dichotoma gum incorporated with Salvia mirzayanii essential oil nanoemulsion. Carbohydr. Polym..

[49] Li Y., Jiang Y., Liu F., Ren F., Zhao G., Leng X. (2011). Fabrication and characterization of TiO_2_/whey protein isolate nanocomposite film. Food Hydrocoll..

[50] Bhatia S., Shah Y.A., Al-Harrasi A., Jawad M., Dıblan S., Khan T.S., Koca E., Aydemir L.Y. (2024). Gelatin/calcium-caseinate films loaded with petitgrain essential oil for sustainable food packaging. Int. J. Food Sci. Technol..

[51] Zhou Y., Wu X., Chen J., He J. (2021). Effects of cinnamon essential oil on the physical, mechanical, structural and thermal properties of cassava starch-based edible films. Int. J. Biol. Macromol..

[52] Huang X., Luo X., Liu L., Dong K., Yang R., Lin C., Song H., Li S., Huang Q. (2020). Formation mechanism of egg white protein/κ-Carrageenan composite film and its application to oil packaging. Food Hydrocoll..

[53] Lam B., How Y., Pui L. (2023). Incorporation of *Bifidobacterium breve* in sodium caseinate-edible film: Physicochemical properties, viability, and antibacterial activity. J. Food Saf..

[54] Lau A., Sarbon N. (2022). Effect of glycerol concentrations on the mechanical and physical properties of chicken skin gelatin-tapioca starch composite films. Food Res..

[55] Wakai M., Almenar E. (2015). Effect of the presence of montmorillonite on the solubility of whey protein isolate films in food model systems with different compositions and pH. Food Hydrocoll..

[56] Khotsaeng N., Simchuer W., Imsombut T., Srihanam P. (2023). Effect of Glycerol Concentrations on the Characteristics of Cellulose Films from Cattail (*Typha angustifolia* L.) Flowers. Polymers.

[57] Farahnaky A., Saberi B., Majzoobi M. (2013). Effect of Glycerol on Physical and Mechanical Properties of Wheat Starch Edible Films. J. Texture Stud..

[58] Hassannia-Kolaee M., Khodaiyan F., Shahabi-Ghahfarrokhi I. (2016). Modification of functional properties of pullulan–whey protein bionanocomposite films with nanoclay. J. Food Sci. Technol..

[59] Zolfi M., Khodaiyan F., Mousavi M., Hashemi M. (2014). Characterization of the new biodegradable WPI/clay nanocomposite films based on kefiran exopolysaccharide. J. Food Sci. Technol..

[60] Ge L., Zhu M., Xu Y., Li X., Li D., Mu C. (2017). Development of Antimicrobial and Controlled Biodegradable Gelatin-Based Edible Films Containing Nisin and Amino-Functionalized Montmorillonite. Food Bioproc Tech..

[61] Jaberifard F., Almajidi Y.Q., Arsalani N., Ghorbani M. (2024). A self-healing crosslinked-xanthan gum/soy protein based film containing halloysite nanotube and propolis with antibacterial and antioxidant activity for wound healing. Int. J. Pharm..

[62] Rashidi M.J., Nasiraie L.R., Zomorrodi S., Jafarian S. (2023). Development and characterization of novel active opopanax gum and gelatin bio-nanocomposite film containing zinc oxide nanoparticles and peppermint essential oil. J. Food Meas. Charact..

[63] Namazi H., Hasani M., Yadollahi M. (2019). Antibacterial oxidized starch/ZnO nanocomposite hydrogel: Synthesis and evaluation of its swelling behaviours in various pHs and salt solutions. Int. J. Biol. Macromol..

[64] Das D., Panesar P.S., Saini C.S. (2024). Effect of montmorillonite (MMT) on the properties of soybean meal protein isolate-based nanocomposite film loaded with debittered kinnow peel powder. Food Res. Int..

[65] Morariu S., Brunchi C.-E., Honciuc M., Iftime M.-M. (2023). Development of Hybrid Materials Based on Chitosan, Poly(Ethylene Glycol) and Laponite^®^ RD: Effect of Clay Concentration. Polymers.

[66] Bhatia S., Al-Harrasi A., Shah Y.A., Jawad M., Al-Azri M.S., Ullah S., Anwer K., Aldawsari M.F., Koca E., Aydemir L.Y. (2023). The Effect of Sage (*Salvia sclarea*) Essential Oil on the Physiochemical and Antioxidant Properties of Sodium Alginate and Casein-Based Composite Edible Films. Gels.

[67] Loste J., Lopez-Cuesta J.-M., Billon L., Garay H., Save M. (2019). Transparent polymer nanocomposites: An overview on their synthesis and advanced properties. Prog. Polym. Sci..

[68] Tang S., Zou P., Xiong H., Tang H. (2008). Effect of nano-SiO2 on the performance of starch/polyvinyl alcohol blend films. Carbohydr. Polym..

[69] Wang Y., Ma J., Xu Q., Zhang J. (2017). Fabrication of antibacterial casein-based ZnO nanocomposite for flexible coatings. Mater. Des..

[70] Oymaci P., Altinkaya S.A. (2016). Improvement of barrier and mechanical properties of whey protein isolate based food packaging films by incorporation of zein nanoparticles as a novel bionanocomposite. Food Hydrocoll..

[71] Azevedo V.M., Dias M.V., Borges S.V., Costa A.L.R., Silva E.K., Medeiros É.A.A., Soares N.d.F.F. (2015). Development of whey protein isolate bio-nanocomposites: Effect of montmorillonite and citric acid on structural, thermal, morphological and mechanical properties. Food Hydrocoll..

[72] Müller K., Jesdinszki M., Schmid M. (2017). Modification of Functional Properties of Whey Protein Isolate Nanocomposite Films and Coatings with Nanoclays. J. Nanomater..

[73] Alizadeh-Sani M., Kia E.M., Ghasempour Z., Ehsani A. (2021). Preparation of Active Nanocomposite Film Consisting of Sodium Caseinate, ZnO Nanoparticles and Rosemary Essential Oil for Food Packaging Applications. J. Polym. Environ..

[74] Nakagaki S., Machado G.S., Stival J.F., dos Santos E.H., Silva G.M., Wypych F. (2021). Natural and synthetic layered hydroxide salts (LHS): Recent advances and application perspectives emphasizing catalysis. Prog. Solid State Chem..

[75] de Azeredo H.M.C. (2009). Nanocomposites for food packaging applications. Food Res. Int..

[76] Seray M., Skender A., Hadj-Hamou A.S. (2021). Kinetics and mechanisms of Zn^2+^ release from antimicrobial food packaging based on poly (butylene adipate-co-terephthalate) and zinc oxide nanoparticles. Polym. Bull..

[77] Venkatesan R., Rajeswari N. (2019). Nanosilica-reinforced poly(butylene adipate-co-terephthalate) nanocomposites: Preparation, characterization and properties. Polym. Bull..

[78] Sharma R., Jafari S.M., Sharma S. (2020). Antimicrobial bio-nanocomposites and their potential applications in food packaging. Food Control.

[79] Picchio M.L., Linck Y.G., Monti G.A., Gugliotta L.M., Minari R.J., Igarzabal C.I.A. (2018). Casein films crosslinked by tannic acid for food packaging applications. Food Hydrocoll..

[80] Gontard N., Guilbert S., Cuq J. (1992). Edible Wheat Gluten Films: Influence of the Main Process Variables on Film Properties using Response Surface Methodology. J. Food Sci..

[81] ASTM (2015). Standard Test Method for Transparency of Plastic Sheeting. Annual Book of ASTM Standards-Plastics (I).

[82] Zhao J., Wang Y., Liu C. (2022). Film Transparency and Opacity Measurements. Food Anal. Methods.

[83] (2018). Test Method for Tensile Properties of Thin Plastic Sheeting.

[84] Aouadi A., Saud D.H., Rebiai A., Achouri A., Benabdesselam S., El-Mordy F.M.A., Pohl P., Ahmad S.F., Attia S.M., Abulkhair H.S. (2024). Introducing the antibacterial and photocatalytic degradation potentials of biosynthesized chitosan, chitosan–ZnO, and chitosan–ZnO/PVP nanoparticles. Sci. Rep..

[85] Sarhadi H., Shahdadi F., Sardoei A.S., Hatami M., Ghorbanpour M. (2024). Investigation of physio-mechanical, antioxidant and antimicrobial properties of starch–zinc oxide nanoparticles active films reinforced with Ferula gummosa Boiss essential oil. Sci. Rep..

[86] Lumivero XLSTAT Statistical and Data Analysis Solution. https://www.xlstat.com.

